# Impact of Astroprincin (FAM171A1) Expression in Oral Tongue Cancer

**DOI:** 10.3389/froh.2020.599421

**Published:** 2020-11-05

**Authors:** Awais Wahab, Alhadi Almangush, Leif C. Andersson, Pentti Nieminen, Tuula Salo

**Affiliations:** ^1^Department of Pathology, University of Helsinki, Helsinki, Finland; ^2^Department of Oral and Maxillofacial Diseases, University of Helsinki, Helsinki, Finland; ^3^Research Program in Systems Oncology, Faculty of Medicine, University of Helsinki, Helsinki, Finland; ^4^Institute of Biomedicine, Pathology, University of Turku, Turku, Finland; ^5^Faculty of Dentistry, University of Misurata, Misurata, Libya; ^6^Medical Informatics and Statistics Research Group, University of Oulu, Oulu, Finland; ^7^Cancer and Translational Medicine Research Unit, Medical Research Centre Oulu, University of Oulu and Oulu University Hospital, Oulu, Finland; ^8^Translational Immunology Research Program (TRIMM), University of Helsinki, Helsinki, Finland

**Keywords:** oral tongue squamous cell carcinoma (OTSCC), recurrence, tumor progression, astroprincin (APCN), FAM171A1

## Abstract

Astroprincin (APCN, FAM171A1) is a recently characterized transmembrane glycoprotein that is abundant in brain astrocytes and is overexpressed in some tumors. However, the expression and role of APCN is unknown in oral tongue squamous cell carcinoma (OTSCC). Aim of this study was to investigate the expression of APCN in OTSCC tissue samples and to analyze possible association of APCN with clinicopathological features and survival rates. This study included 76 patients treated for OTSCC. Expression of APCN in OTSCC tissue sections was examined by immunohistochemistry with a rabbit polyclonal antibody (MAP346) against APCN. All tumors were scored for intensity and percentage of APCN staining at the superficial, middle, and invasive front areas. High expression of APCN was significantly associated with increased tumor size (*P* = 0.013) and with OTSCC recurrence (*P* = 0.026). In this pilot study, we observed that the amount of APCN is associated with the size and recurrence of OTSCC. This finding suggests a role of APCN during OTSCC progression.

## Introduction

Oral squamous cell carcinoma (OSCC) is a highly prevalent epithelial malignancy of the oral cavity and afflicts over 300,000 people annually [1]. Early detection and location of the tumor for OSCC has been associated with overall survival rate [2]. Oral tongue squamous cell carcinoma (OTSCC) is generally presented with aggressive clinical behavior and worse prognosis than other sites of the oral cavity [3]. The rationale for this is rich lymphatic network and muscularized structure of the tongue that accelerates invasion and lymph node metastasis [3]. Recent studies have indicated rise in the incidence of OTSCC among younger men [4]. The overall incidence of OTSCC has also increased and constitute 25–40% of oral carcinomas [3, 5]. Recent research in OTSCC has primarily focused to explore new biomarkers and additional risk factors [6].

Despite the progress in diagnosis and treatment of OTSCC, improvement in survival rates among patients with advanced stages have remained modest [7]. The prognosis of oral cancer is commonly estimated through clinical TNM classification [8]. However, the prognosis and response to treatment often deviate considerably between patients with the same TNM stage [9, 10]. Although numerous molecular prognostic biomarkers have been assessed in the last decade, none are currently in clinical pathology practice [11, 12]. Therefore, there is still a need to identify reliable markers that provide prognostic evaluation of OTSCC to facilitate treatment planning.

Rasila et al. [13] recently characterized a novel endogenous protein called astroprincin (APCN, also known as FAM171A1) that is abundant in brain astrocytes. APCN is an evolutionarily conserved 98-kDa transmembrane type I glycoprotein. APCN is expressed in placental trophoblasts, kidneys, pancreas and heart muscle [13]. APCN is also expressed in some malignancies, such as nodular melanoma and lobular breast cancer [13]. Higher APCN expression was seen in the tumor invasive front than in the intra-tumoral cells [13]. The authors showed that upregulated APCN expression induced an invasive growth pattern, with sprouting of slender-like projections in melanoma cells [13]. Furthermore, FAM171A1 (APCN) expression has also recently been shown to correlate with invasive phenotypes and poor overall survival in triple-negative breast cancers [14].

Our present study was conducted to investigate the expression of APCN in OTSCC tissue samples and to analyze the possible association between APCN and clinicopathological features and survival rates of OTSCC patients.

## Materials and Methods

### Patients and Clinical Data

This study included 114 patients with OTSCC. Histopathological data of the patients collected from the pathology reports included tumor grade (grade I-III). Clinical data obtained from the department of otorhinolaryngology (Oulu University Hospital) included age, sex, status of the patient (alive or deceased), cause of death, follow-up time (months), clinical stage of the tumor (cT), and lymph node status (cN). Out of the original 114 cases, 38 were excluded due to incomplete clinical data available. The number of included OTSCC cases in the final analyses was thus 76.

### Astroprincin Immunohistochemistry

Tumor paraffin blocks of 114 postoperative resected OTSCC cases were collected from the pathology department. Tissue sections of 4-μm thickness were mounted on slides and allowed to dry overnight at 37°C. The slides were subjected to deparaffinization and rehydration. The primary polyclonal antibody MAP346 was produced as described by Rasila et al. [13] MAP346 was diluted 1:750 and incubated for 15 min. Staining was performed as previously described by Rasila et al. [13] using Bond Polymer Refine Detection kit and Leica Bond RX staining automat. Dako's rabbit negative control X0903 was first used in the same concentration as antibody dilution. For the negative control, the primary antibody was omitted, and for the positive control was breast cancer tissue sections which have previously shown to be positive for MAP346 (Rasila et al. [13]).

### Evaluation of Astroprincin Immunoexpression

APCN immunoexpression was examined with a staining reaction of light or dark brown color in the cytoplasm and nuclei of OTSCC cells. The dark brown staining was considered high immunoexpression and light brown staining was considered low expression. The immunoexpression between high and low was considered moderate. For evaluation, both nuclear and cytoplasmic expression were analyzed and the average fraction of stained cancer cells was calculated.

**Figure 1:**
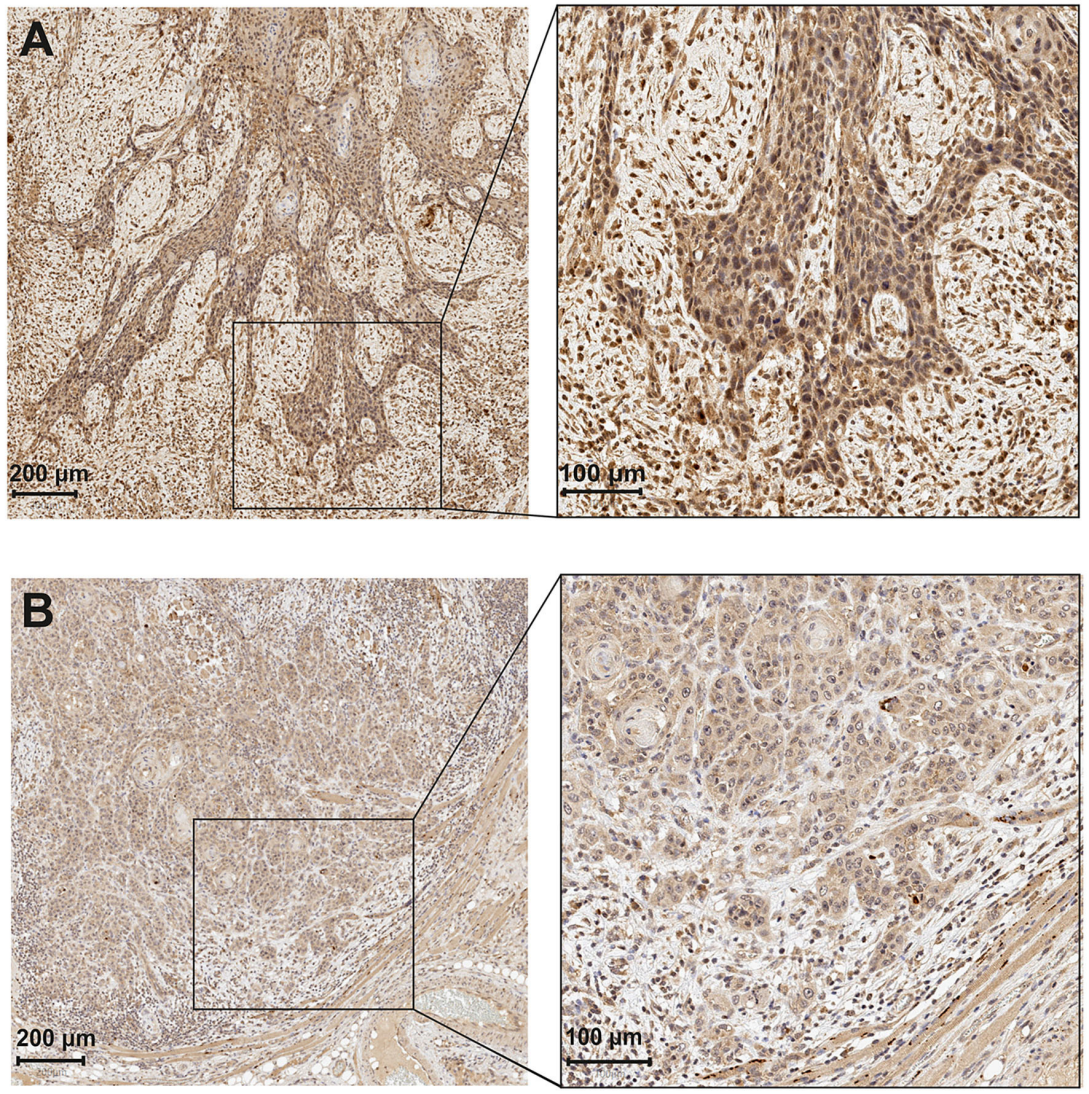
Astroprincin (APCN) expression in OTSCC at the invasive front. **(A)** An example of high percentage (>85%) and intensity at invasive front. **(B)** low percentage (<85%) and intensity at invasive front.

For scoring, all slides were classified into the following two main factors based on the proportion of positively stained cancer cells by the APCN antibody: intensity and percentage of staining by APCN for OTSCC specimens. All tumors were divided into three areas for evaluation of APCN, namely (1) superficial, (2) middle, and (3) invasive front. Each of the three areas were categorized into the following three scores based on APCN staining intensity: low intensity (scored 1), moderate intensity (scored 2), and high intensity (scored 3). Different cut-off points (10, 20, 30, 50, and 85%) were used to evaluate the association of APCN percentage score with clinicopathologic characteristics. The distribution of stained tumor by APCN for each of three areas were assessed on the basis of most relevant percentage (<85% and >85%) for low and high percentage score (Figure 1).

Two investigators (AW and AA) evaluated all scanned specimens independently with QuPath software Version 0.1.2, which is an open-source software for digital pathology image analysis. In case of disagreement, the samples were re-evaluated to determine a consistent final score. The Kappa coefficient of agreement between the observers was 0.773. Observers were blinded to all clinical data.

### Statistical Analysis

The significance level was observed higher at the invasive front than the other two regions, and therefore it was selected for all statistical analyses in relation to clinicopathological variables and survival analyses. The association between clinicopathological characteristics (age, gender, grade, stage, cT, cN, and recurrence) and the expression of APCN intensity and percentage at the invasive front was analyzed with cross-tabulation.

**Figure 2:**
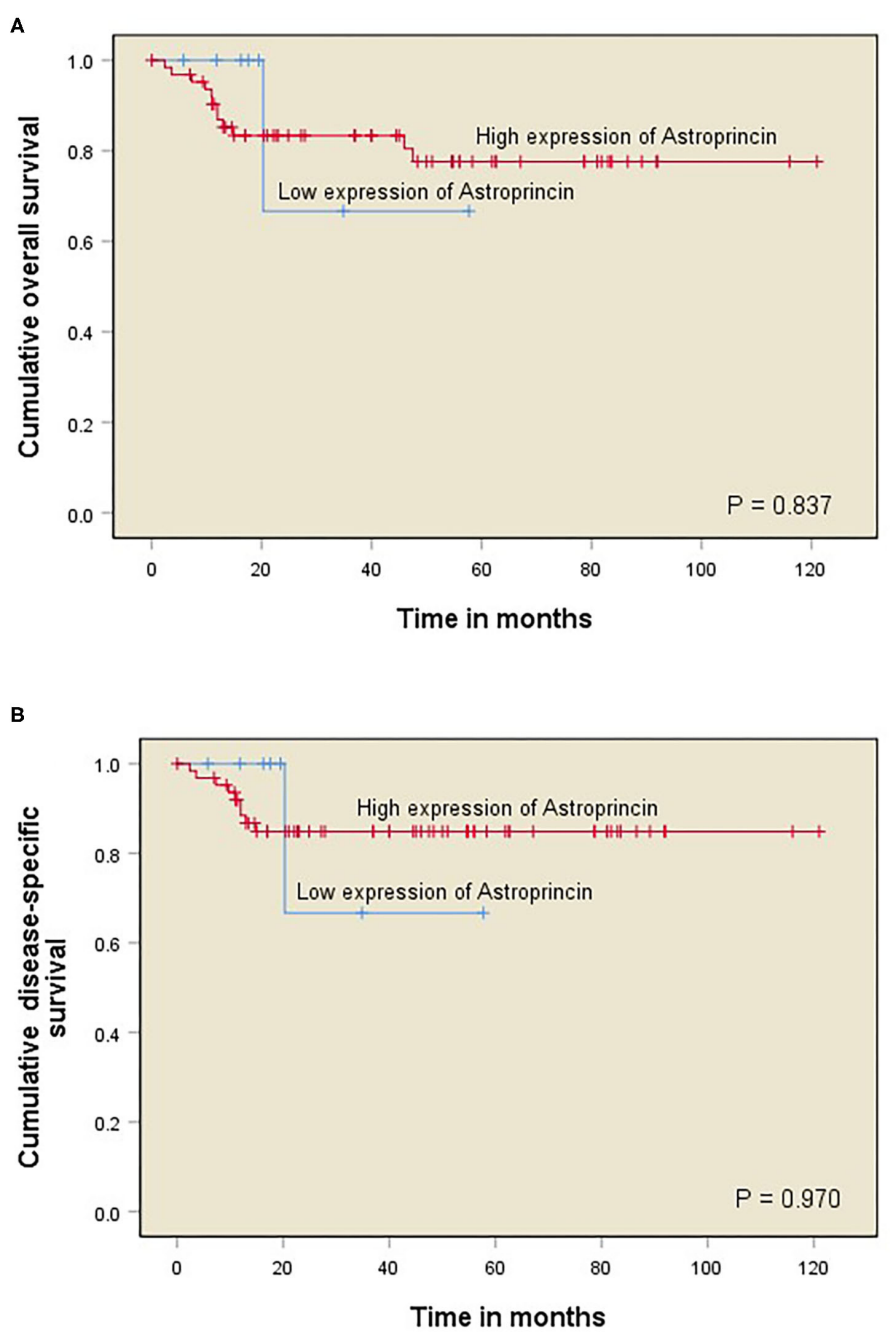
Kaplan–Meier curves of OTSCC patient survival divided into two groups corresponding to immunoexpression of APCN at 85% cut-off point (<85% vs. >85%). Overall survival (OS) for percentage of APCN at the invasive front **(A)**; Disease-specific survival (DSS) for percentage of APCN at the invasive front **(B)**.

The survival analyses performed included overall survival (OS; refers to the time from surgery to death or last follow-up) and disease-specific survival (DSS; refers to the time from surgery to death from OTSCC or last follow-up). OS and DSS curves were constructed with the Kaplan–Meier method. A log-rank test was used to compare the statistical significance between the APCN groups (Figure 2). Cox proportional hazard models were used to obtain the unadjusted hazard ratios (HRs) and their 95% confidence intervals (CIs). All statistical analyses were performed with IBM SPSS Statistics (version 24) software.

## Results

Forty-five (59.21%) of the OTSCC patients were males and 31 (40.79%) were females (Table 1). Early-stage (T1 and T2) tumors were observed in 57 (75%) patients and late-stage (T3 and T4) tumors in 19 (25%) patients (Table 1). The median follow-up period was 35.8 months and range was 0-121 months.

**Table 1:** Clinicopathological characteristics of 76 patients with OTSCC in relation to high and low percentage of APCN at the invasive front.

**Variable**	**Total**	**High APCN percentage at invasive front**	**Low APCN percentage at invasive front**	***P*-value of chi-square test**
	**Total number of cases *N* = 76**	**Number (%) 68 (89.5%)**	**Number (%) 8 (10.5%)**	
Age				0.220
<60	24 (31.6%)	23 (95.8%)	1 (4.2%)	
> 60	52 (68.4%)	45 (86.5%)	7 (13.5%)	
Gender				0.085
Female	31 (40.79%)	30 (96.8%)	1 (3.2%)	
Male	45 (59.21%)	38 (84.45%)	7 (15.55%)	
WHO Grade				0.630
I	16 (21.05%)	15 (93.75%)	1 (6.25%)	
II	45 (59.21%)	39 (86.7%)	6 (13.30%)	
III	15 (19.74%)	14 (93.3%)	1 (6.7%)	
cTNM Stage				0.084
Early stage (I and II)	57 (75%)	53 (92.98%)	4 (7.02%)	
Late stage (III and IV)	19 (25%)	15 (78.95)	4 (21.05%)	
cT				**0.013**
1	28 (36.8%)	26 (92.86%)	2 (7.14%)	
2	34 (44.7%)	32 (94.1%)	2 (5.9%)	
3	10 (13.15%)	6 (60%)	4 (40%)	
4	4 (5.35%)	4 (100%)	0	
cN				0.724
0	57 (75%)	51 (89.47%)	6 (10.53%)	
1	14 (18.4%)	13 (92.85%)	1 (7.15%)	
2	5 (6.6%)	4 (80%)	1 (20%)	
Recurrence^*^				**0.026**
No	44 (64.70%)	36 (81.81%)	8 (18.19%)	
Yes	24 (35.30%)	24 (100%)	0	

*Clinical stage of the tumour (cT), and lymph node status (cN)*.

* *Recurrence data of the eight cancer cases were not available. P values in bold are statistically significant*.

### Relationship Between APCN Expression, Clinicopathologic Characteristics, and Survival

We observed a significant association between tumor size and a high percentage of APCN expression at the tumor invasive front (*P* = 0.013). Enhanced expression of APCN at the invasive front was significantly associated with disease recurrence in the cross-tabulation analysis (*P* = 0.026; Table 1). We did not observe a significant relationship between APCN expression intensity and any other clinicopathological characteristics (*P* > 0.05). In univariable analysis (Table 2), APCN expression did not have prognostic significance for OS (HR 1.24, 95% CI 0.16–9.60; *P* = 0.840) or for DSS (HR 1.04, 95% CI 0.132–8.24; *P* = 0.970), (Figure 2).

**Table 2:** Survival analyses of 76 cases with OTSCC.

**Variable**	**Overall survival (OS)**	**Disease-specific survival (DSS)**
	**HR (95% CI)**	**HR (95% CI)**
Percentage of APCN at IF		
Low (<85%)	1	1
High (>85%)	1.24 (0.16–9.60)	1.04 (0.132–8.24)
	*P* = 0.840	*P* = 0.970
Intensity of APCN at IF		
Low	1	1
Moderate	0.83 (0.10–6.90)	0.724 (0.08–6.23)
	*P* = 0.860	*P* = 0.770
High	0.30 (0.035–2.65)	0.285 (0.03–2.56)
	*P* = 0.280	*P* = 0.260
Age		
≤ 60	1	1
>60	1.76 (0.48–6.40)	1.20 (0.31–4.65)
	*P* = 0.390	*P* = 0.790
Gender		
Female	1	1
Male	0.39 (0.12–1.19)	0.43 (0.12–1.55)
	*P* = 0.100	*P* = 0.200
cTNM Stage		
I-II	1	1
III-IV	0.85 (2.33–3.10)	0.71 (0.15–3.36)
	*P* = 0.800	*P* = 0.670
Grade (WHO)		
I	1	1
II	1.64 (0.34–7.74)	2.31 (0.27–19.23)
	*P* = 0.530	*P* = 0.440
III	2.34 (0.39–14.05)	4.38 (0.45–42.20)
	*P* = 0.350	*P* = 0.200
cT		
T1	1	1
T2	1.31 (0.38–4.51)	0.94 (0.25–3.50)
	*P* = 0.660	*P* = 0.925
T3	0.70 (0.08–6.34)	0.72 (0.080–6.42)
	*P* = 0.760	*P* = 0.765
T4	2.51 (0.28–22.65)	NA
	*P* = 0.410	NA
cN		
N0	1	1
N1	0.77 (0.165–3.56)	1.18 (0.24–5.85)
	*P* = 0.735	*P* = 0.840
N2	2.90 (0.6213.45)	4.05 (0.81–20.13)
	*P* = 0.175	*P* = 0.085
Recurrence^*^		
No	1	1
Yes	2.19 (0.67–7.19)	2.35 (0.632–8.77)
	*P* = 0.195	*P* = 0.200

*The overall survival and disease specific analyses present the prognostic significance of Astroprincin (APCN) at the invasive front (IF) and other clinicopathologic variables*.

* *Recurrence data of the eight cancer cases were not available*.

*IF, invasive front. NA, not available*.

## Discussion

APCN has emerged evolutionarily as a highly conserved type 1 transmembrane glycoprotein that regulates cell cytoskeletal dynamics and tumor cell invasion [13]. Elevated expression of APCN has been reported in lobular and triple-negative breast cancer cells and at the invasive front of nodular melanoma [13, 14]. Here we investigated for the first time APCN expression in OTSCC and observed that it was associated with tumor size and recurrence. However, the median follow-up time was only 35.8 months and there were missing values of recurrence which are the main limitations in this study.

All cases in the cohort of our study had been treated before 2017 and lacked information on extra-nodal extension of many cases which is part of the N-category in the 8th edition of American Joint Committee on Cancer (AJCC). Therefore, cohort of this study was staged according to the 7th edition of (AJCC).

Few studies have evaluated APCN in different tumors [13, 15]. We hypothesized that APCN might have a role in the progression of OTSCC. Although we observed a significant association between APCN expression and increased tumor size (cT), there was no significant difference in the mortality between patients with low and high APCN expression. However, higher expression of APCN at the invasive front was associated with an increased recurrence rate (*P* = 0.026). In contrast to our study, a high APCN expression level was associated with invasiveness and also poor OS in triple-negative breast cancer cells [15]. The same study also revealed that estrogen receptor alpha regulated APCN expression in the breast cancer cells and that the *APCN* gene was associated with proliferation and migration of triple-negative breast cancer cells. Although its biological function is not yet well-understood, APCN is present in astrocytes and is assumed to be involved in brain development. Bao et al. speculated that since APCN is expressed in the brain tissue, it may explain the frequent tendency of triple-negative breast cancer cells to metastasize into brain tissue [14].

The APCN gene has seven short exons at the extracellular region [13]. The exon seven runs from outside to inside the transmembrane stretch that is highly similar from zebra fish to human. A long intracytoplasmic portion constitute two thirds of the protein, specifically encoded by the large exon 8, which also has many evolutionarily conserved regions [13]. As APCN is involved in the regulation of actin cytoskeletal dynamics, APCN is related to cell shape, cell sprouting, and invasive growth of tumor cells [13]. The evidence of organized actin cytoskeleton maintenance by the APCN is further supported by the disappearance of actin stress fibers after downregulated expression of endogenous APCN. Moreover, complete absence of endogenous APCN resulted in disruption of actin dynamics, which is required for cytokinesis [13]. Therefore, the presence of APCN protein in the invasive front of OTSCC is most probably also related to the invasive progression of tumor. Further investigations are required to unravel its molecular details and role in carcinogenesis.

In conclusion, we have shown that APCN is immunohistologically detected in a set of OTSCC samples, and a high amount of immunoreactive protein is related to tumor size and recurrence. However, in this pilot study, there was no prognostic value of APCN for OS or DSS of 76 patients. Further evaluation of APCN in large multicenter cohorts of OTSCC may be considered to determine whether APCN can be used for OTSCC prognostication.

## Data Availability Statement

The raw data supporting the conclusions of this article will be made available by the authors, without undue reservation.

## Ethics Statement

The studies involving human participants were reviewed and approved by this study was approved by Oulu University hospital and the Finnish National Supervisory authority for Welfare and health (VALVIRA). The patients/participants provided their written informed consent to participate in this study.

## Author Contributions

AW designed the project, carried out the scoring, performed the statistical analysis, interpreted the results, and wrote the manuscript. AA designed the project, carried out the scoring, interpreted the results, and wrote the manuscript. LA contributed in the manuscript text. PN contributed in the statistical part of the analyses. TS designed the project, interpreted results, contributed in the manuscript writing, and supervised the project. All authors critically revised the manuscript.

## Conflict of Interest

The authors declare that the research was conducted in the absence of any commercial or financial relationships that could be construed as a potential conflict of interest. The reviewer RF declared a past co-authorship with one of the authors TS to the handling editor.
